# Maternal Size and Age Shape Offspring Size in a Live-Bearing Fish, *Xiphophorus birchmanni*

**DOI:** 10.1371/journal.pone.0048473

**Published:** 2012-11-06

**Authors:** Holly K. Kindsvater, Gil G. Rosenthal, Suzanne H. Alonzo

**Affiliations:** 1 Department of Ecology and Evolutionary Biology, Yale University, New Haven, Connecticut, United States of America; 2 Centro de Investigaciones Científicas de las Huastecas “Aguazarca”, Calnali, Hidalgo, Mexico; 3 Department of Biology, Texas A&M University, College Station, Texas, United States of America; California State University Fullerton, United States of America

## Abstract

Many studies of offspring size focus on differences in maternal investment that arise from ecological factors such as predation or competition. Classic theory predicts that these ecological factors will select for an optimal offspring size, and therefore that variation in a given environment will be minimized. Yet recent evidence suggests maternal traits such as size or age could also drive meaningful variation in offspring size. The generality of this pattern is unclear, as some studies suggest that it may represent non-adaptive variation or be an artifact of temporal or spatial differences in maternal environments. To clarify this pattern, we asked how maternal size, age and condition are related to each other in several populations of the swordtail *Xiphophorus birchmanni*. We then determined how these traits are related to offspring size, and whether they could resolve unexplained intra-population variation in this trait. We found that female size, age, and condition are correlated within populations; at some of these sites, older, larger females produce larger offspring than do younger females. The pattern was robust to differences among most, but not all, sites. Our results document a pattern that is consistent with recent theory predicting adaptive age- and size-dependence in maternal investment. Further work is needed to rule out non-adaptive explanations for this variation. Our results suggest that female size and age could play an under-appreciated role in population growth and evolution.

## Introduction

Smith and Fretwell [1] predicted that maternal fitness is maximized at a single intermediate level of effort (usually size) per offspring, and that females with additional resources will invest in fecundity, rather than making larger offspring [1]–[3]. A remarkable body of research rooted in this perspective has demonstrated that offspring sizes vary predictably among populations that differ in ecological factors such as predation, density, or acidity [4]–[6]. As a result, ecological factors are viewed as the principle drivers of offspring size [2]–[3]. However, recent empirical evidence has suggested that offspring size can vary with female characteristics as well, leading to potentially meaningful intra-population variation in this trait [7]–[9]. Although intriguing, the importance of these female effects on offspring size is currently debated. Until recently, there was scant theory that could generally explain why selection would favor size- or age-dependent maternal investment [10]–[15]. In addition, size- and age-dependence in maternal investment is difficult to clearly demonstrate without potential confounds because of covariation among multiple life-history traits [13].

Maternal size and age have been related to offspring size in a broad variety of taxa, including aquatic and marine invertebrates [16]–[19], fish [9] [20]–[26], and birds [27]–[28]. Initially, theory addressing this pattern suggested that sibling competition [29] or physiological constraints on offspring investment [30] were needed to explain the correlation between maternal traits and offspring size. Although they are promising explanations for some taxa, these mechanisms are limited to species with density-dependent sibling competition, or specific physiology that links maternal size and offspring size, such as size-dependence in nutrient transfer rate (a mechanism proposed for plants [30]). Two alternative mechanisms for the dependence of offspring size on maternal age and size have been proposed [14]–[15]. First, offspring size is predicted to depend on maternal age when reproductive effort is costly to maternal survival [14]. If young females reduce reproductive effort to maximize survival, selection can also favor smaller offspring depending on the differential fitness benefits of investing in size or number [14]. Second, maternal size is predicted to affect offspring size of livebearers when mortality is greater for small females than for juveniles [15]. Small, livebearing females in this scenario are predicted to produce small offspring, given that they will develop more quickly, and thus be born sooner, than large offspring. By producing small offspring, these females minimize the amount of time the developing offspring are exposed to the maternal risk of mortality.

Although these theoretical advances are encouraging, clearly demonstrating maternal size or age effects on offspring size remains difficult. One problem is that female size and age are themselves often strongly correlated, making their individual effects difficult to distinguish [13], especially as age is often not measured directly. Furthermore, the effect of maternal traits on offspring size may be obscured by variation in maternal condition [31]–[34]. Finally, in natural populations, removing temporal or spatial variation in the maternal environment has proven difficult [13]. Despite these problems, there is some evidence that size-dependence in maternal investment is robust to ecological differences [9].

Here, we examine how maternal size, age, and condition affect offspring size in wild populations. We first ask how maternal traits covary within and among populations. We then determine how offspring size and number were related to these traits, and if differences in ecological context affected the relationship between reproductive traits and maternal traits. According to the theory described above, livebearing fish that experience survival costs of reproduction are likely to show age- or size-dependence [14]–[15]. We therefore chose to study these traits in wild populations of the livebearing swordtail *Xiphophorus birchmanni*. This species is ideal for our aim of examining the relationship between maternal traits and offspring size and number because costs of reproduction that decrease female swimming performance (and presumably survival) have been shown in related species [35]. Furthermore, reproductive investment of *X. birchmanni* can be quantified prior to parturition in gravid females, as embryo dry mass has been found to be strongly related to offspring size at birth in poeciliids [36]. Thus, we expect that female size, age, or condition will be associated with egg size in these fish in a variety of ecological settings.

## Methods

### Ethics Statement

Our methods were vetted and approved by the Yale Institutional Animal Care and Use Committee (protocol number 2007–10908*;* renewed for 2008 and 2010).

### Field Methods

We studied multiple populations of *X. birchmanni* in the Mexican state of Hidalgo. Animals were collected with permission from the Mexican government (Permiso de Pesca de Fomento No. DGOPA.07311.130709.2261). Female sizes and ages, and corresponding offspring sizes and numbers, were measured at sites where *X. birchmanni* is abundant; *X. birchmanni* is not endangered or threatened. Our sites were all in public waterways. We initially chose two sites (a main channel and a tributary site) with large differences in the size ranges of mature fish. Despite observed differences in size, previous work found these sites were genetically similar at neutral markers [37]. In 2008 and 2010, fish were collected from these sites: the main river channel at San Pedro (20.950N, 98.523W), and a second population in a tributary more than two km upstream (Cocalaco, 20.958N, 98.521W). In 2010, a third population in a different river with an intermediate size distribution (Coacuilco, 21.098N, 98.586W) was added. Fish at this site are genetically distinct from the first two sites (Culumber ZW, *unpublished data*). We selected these sites to make sure that size distributions overlapped among sites, but also to ensure that a large range of female sizes was present in our sample.

At each site, we caught females in minnow traps and euthanized them using a solution of MS-222 within two hours of capture. We then measured mature females and removed their gonads. These females were preserved for later aging (described below) or for lipid content analysis (described below). In 2010, all females were preserved for both aging and lipid content analysis. If a female had fertilized embryos in her gonads, they were counted, and the developmental stages of offspring noted (following Stearns, SC, *unpublished data*; [38]). We excluded females with unfertilized embryos from further analysis, as these females could be in the process of yolking eggs. Embryos from a given brood were all the same stage (*i.e.*, females do not superfetate [39]). Three embryos from each female were then weighed individually to obtain a wet weight. These three embryos were then dried at 60° C for more than 48 h and weighed to the nearest 0.0001 g. The mean of the three dry weights was used to estimate maternal investment per offspring, i.e., offspring size.

### Female Age

In order to measure female age, female carcasses were first preserved in ethanol to aid later otolith extraction. After extraction, otoliths were mounted on slides using thermoplastic glue, polished, and photographed; age in days was estimated from daily growth rings. Otoliths were prepared and aged by the Fish Ageing Service (Portarlington, Victoria, Australia).

### Female Condition

Elemental analysis of C:N ratios in female body tissue to quantify variation in female condition. This method has been used to measure condition in aquatic organisms [40]. A pilot study in 2008 showed that lipid estimates from samples of headless females were strongly correlated with lipid estimates from female bodies with intact heads (Pearson’s correlation = 0.97, t = 10.1525, d.f. = 7, *P*<0.001). Based on these data, in 2010 headless female carcasses were dried and processed, and female condition measured [40]. This allowed the simultaneous preservation of the female’s head in ethanol for otolith removal. Dried samples were ground with a SPEX Certiprep 6750 freezer mill (SPEX Certiprep, Metuchen, New Jersey, USA). Elemental analysis was performed with a ThermoFinnigan DeltaPlus Advantage stable isotope mass spectrometer (Thermo Scientific, Waltham, Massachusetts, USA) at the Earth Systems Center for Stable Isotope Studies at the Yale Institute for Biospheric Studies.

### Statistical Analyses

Statistics were done with the R statistical language [41]. We first used ANCOVA to determine how maternal size, age, and condition varied among each site. We combined size data for each year at the sites that were sampled multiply for this analysis. Next, linear regressions were used to examine within-site effects of female size, age, condition, and developmental stage on the two response variables, offspring size and number. As offspring number is expected to increase geometrically with female length, fecundity data were log transformed. Data were analyzed separately by year for the two sites with two years of data. We chose to analyze the relationships between the predictor variables (female size, age, and condition) and response variables separately for each site in each year, as different environmental conditions between years could generate differences in female investment patterns.

**Figure 1:**
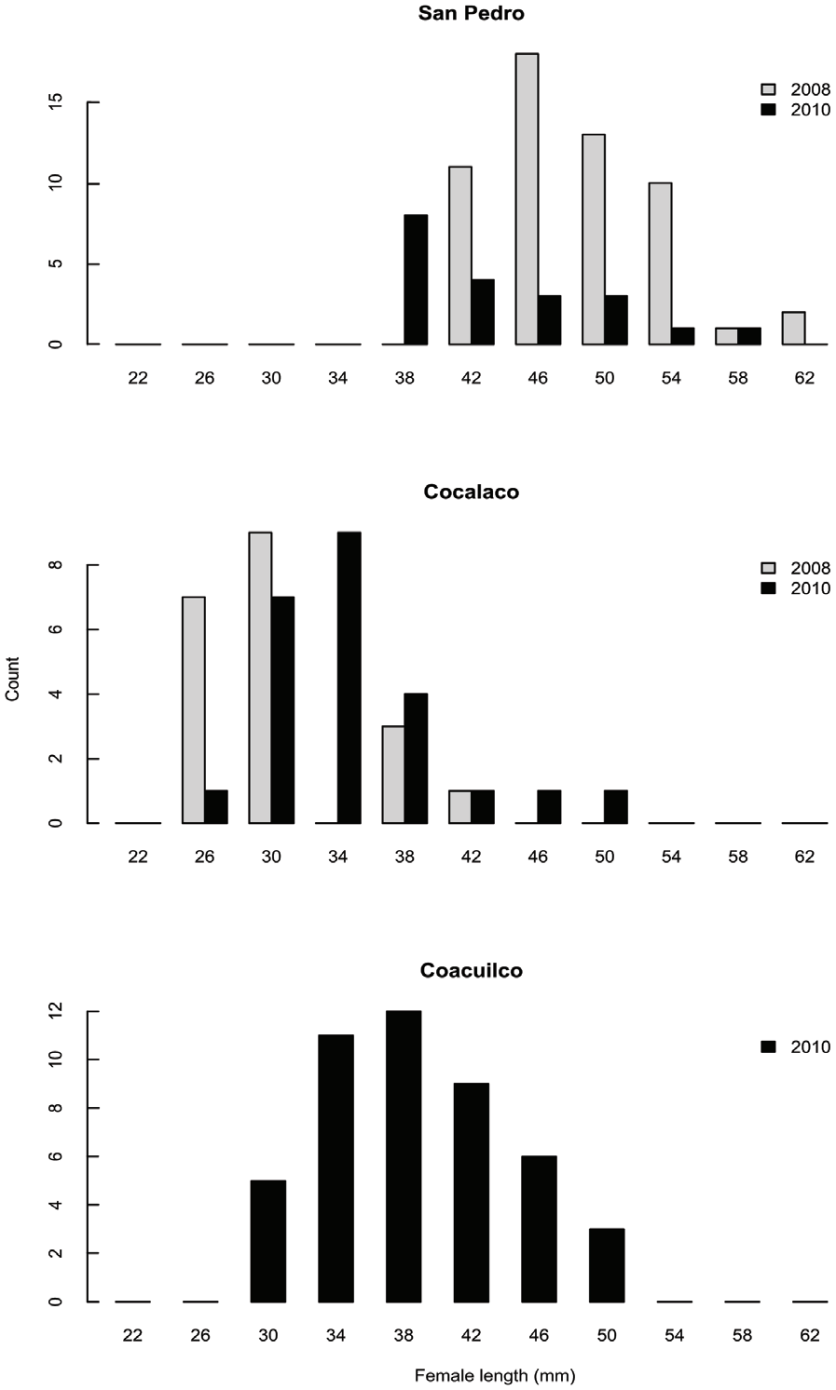
Site and year differences in female sizes (standard length, in mm). Females are mature (i.e., gravid). Top panel: San Pedro. Middle panel: Cocalaco. Bottom panel: Coacuilco. In 2010 we added Coacuilco as a third site to examine females in a size range intermediate to San Pedro and Cocalaco.

## Results

### Covariation of Maternal Traits within and Among Sites and Years

**Figure 2:**
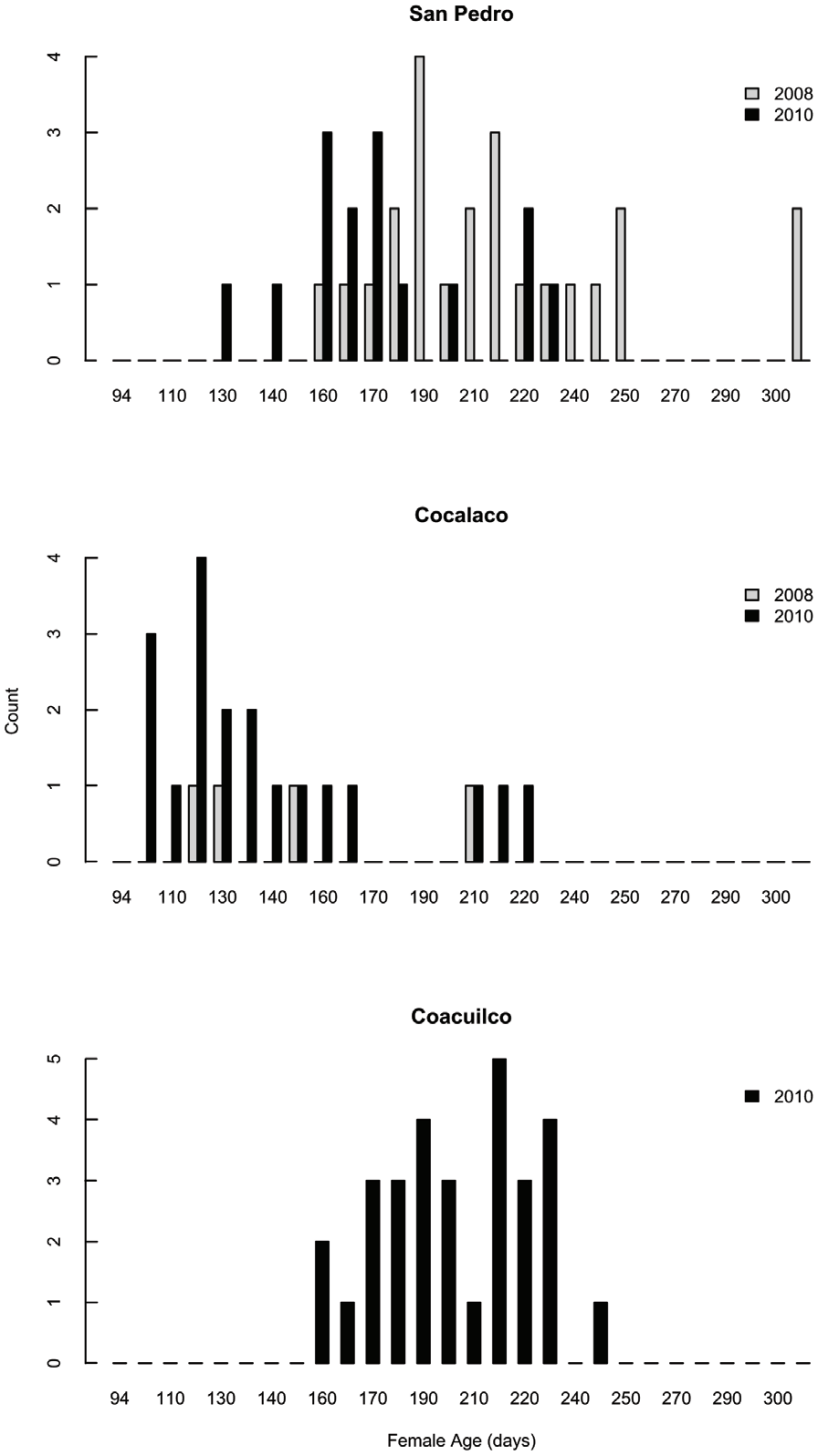
Site and year differences in female age (in days). Females are mature (i.e., gravid). Top panel: San Pedro. Middle panel: Cocalaco. Bottom panel: Coacuilco. In 2010 we added Coacuilco as a third site to examine females in a size range intermediate to San Pedro and Cocalaco.

A major goal of this study was to determine if the relationship between maternal traits and offspring size was robust among years and in different populations. Maternal size (the dependent variable) varied significantly with site and maternal age (data is shown in Figures 1 and 2). There was no significant site by age interaction and no significant year effect, so we excluded these terms from the final model (ANCOVA: F_2, 88_ = 53.9; *P*<0.001). Female size, age, site, and all interactions were significant predictors of female condition (ANCOVA: F_7, 50_ = 7.1; *P*<0.001). This initial analysis suggests that the largest females tended to be the oldest and in the best condition across sites (Figure S1). The differences in adult female size and age among sites reflected differences in size and age at maturity of females in these populations. Figures 1 and 2 show that females matured at a smaller size and younger age at Cocalaco, and that overall the ranges of mature female sizes and ages were smaller at Cocalaco than at San Pedro, suggesting that the ecological factors shaping swordtail life-history traits differed among sites.

### The Relationship between Maternal Phenotype, Offspring Size and Number

**Figure 3:**
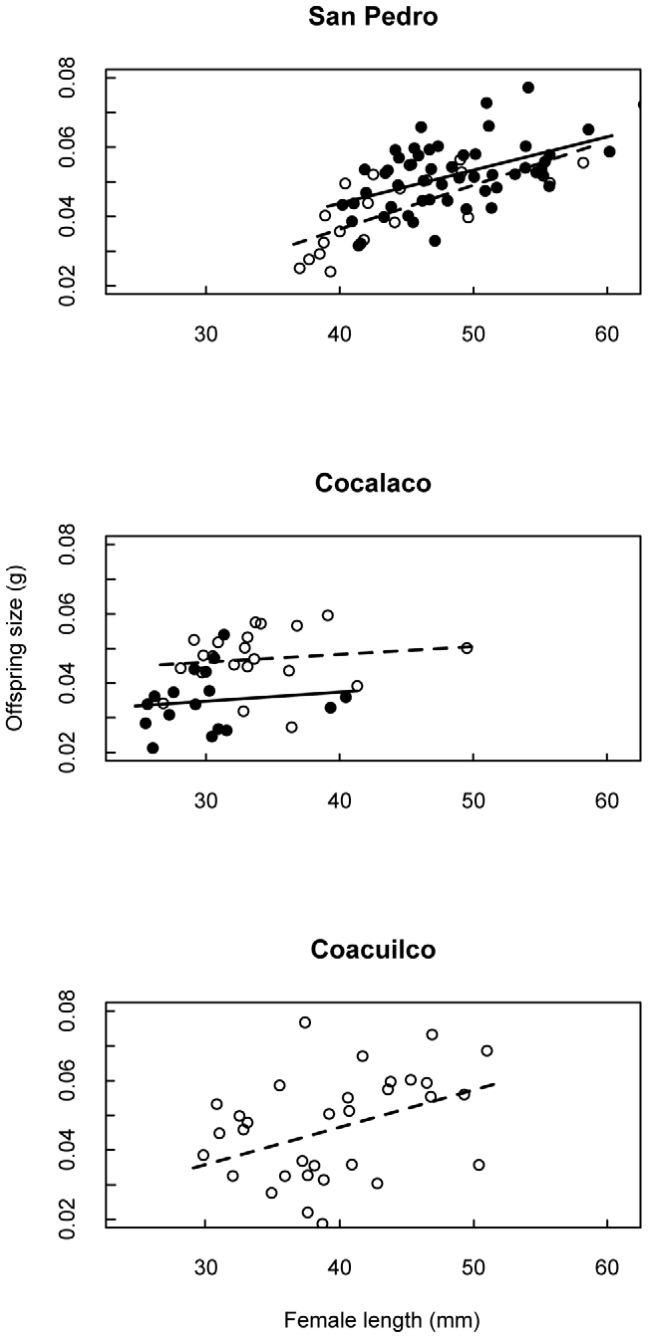
Female size and offspring size. Solid points are data from 2008; open points are 2010. Female length is significantly related to offspring size at San Pedro (top panel) and Coacuilco (bottom panel). The trend at Cocalaco in each year was not significant. A separate linear model was estimated for each year of data at Cocalaco and San Pedro as female size varied significantly with year.

**Figure 4:**
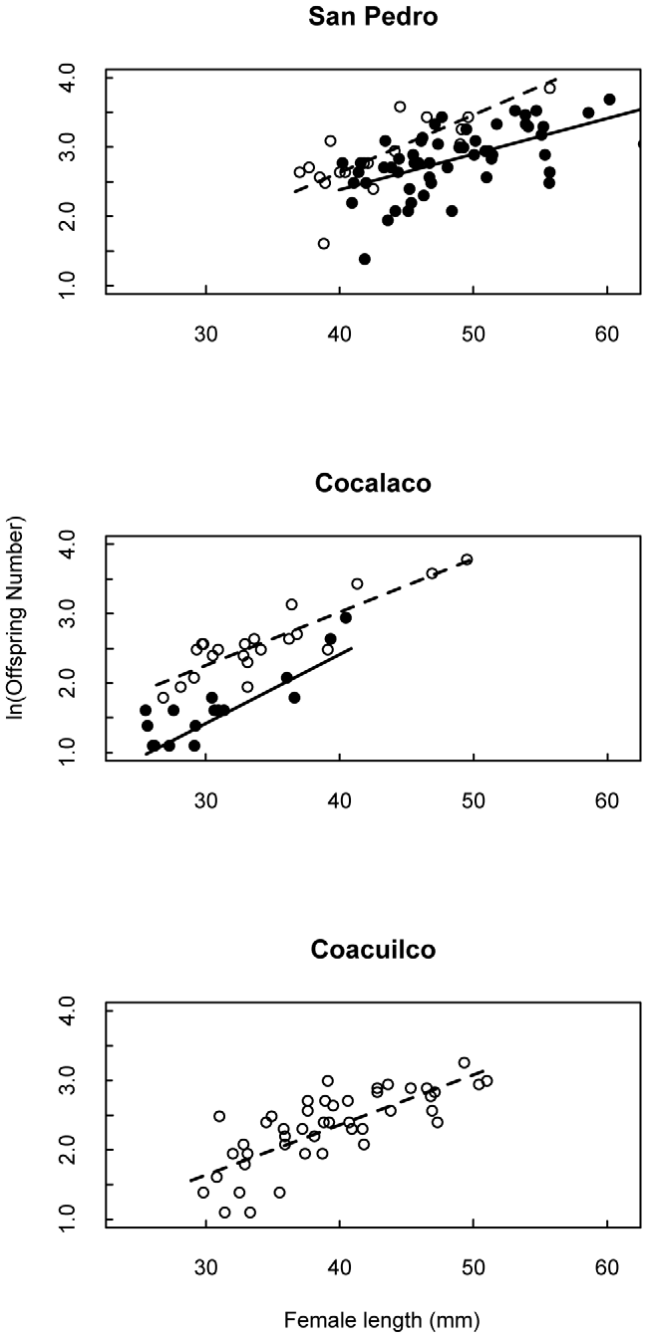
Female size and offspring number are positively correlated for all sites and years; Solid points are data from 2008; open circles are 2010. Offspring number was log-transformed, as fecundity is expected to increase geometrically with female size. In all cases, slopes are significantly different from zero.

Our primary objective was to determine whether maternal size, age, and condition consistently affected investment in offspring size despite these ecological differences. We found female size alone was the best predictor of both offspring size and number at each site (Tables 1, 2; Figures 3, 4); developmental stage, female age, and condition were not significant. Female size was positively correlated with offspring size for both years of data at San Pedro, and for the single year of data at Coacuilco. Although not significant, at Cocalaco (the tributary site) the relationship between female size and offspring size was consistent with the other sites. Consistent with our understanding of fish life histories, female fecundity was positively correlated with female size at all three sites (Table 2; Figure 4).

**Table 1:** Offspring size as a function of female size for each site in each year.

	2008		2010	
	F-stat	P-value	F-stat	P-value
*San Pedro*				
Female size	19.53 _1, 53_	**<0.001**	18.70 _1, 17_	**<0.001**
*Cocalaco*				
Female size	0.30 _1, 15_	0.59	0.34 _1, 19_	0.56
*Coacuilco*				
Female size	n/a	n/a	5.80 _1, 31_	**0.022**

The model of offspring size initially included developmental stage, female age, condition, and size; backwards stepwise removal of non-significant effects revealed maternal size was the best predictor of offspring size (as well as number). Numerator and denominator degrees of freedom are listed as subscripts for each F-statistic. Bold face font indicates significance at *P*<0.05.

**Table 2:** Offspring number as a function of female size for each site in each year.

	2008		2010	
	F-stat	P-value	F-stat	P-value
*San Pedro*				
Female size	27.44 _1, 53_	**<0.001**	38.05 _1, 17_	**<0.001**
*Cocalaco*				
Female size	21.84 _1, 18_	**<0.001**	64.85 _1, 20_	**<0.001**
*Coacuilco*				
Female size	n/a	n/a	44.38 _1, 44_	**<0.001**

The model of offspring number initially included female age, condition, and size; backwards stepwise removal of non-significant effects revealed maternal size was the best predictor of offspring number. Numerator and denominator degrees of freedom are listed as subscripts for each F-statistic. Bold face font indicates significance at *P*<0.05.

## Discussion

Studies of life-history traits have traditionally focused on understanding differences in offspring size among populations [4]. However, recent research has revealed that meaningful variation in offspring size may exist within populations, and can be related to maternal size and age (e.g. [9]). We found a positive relationship between offspring size and maternal size at two of our sites, and at one of these sites in two years. Our study demonstrates that larger, older swordtails produce larger offspring. We found this pattern in females from two populations with varying size and age ranges. However, we did not detect size- or age-dependence in maternal investment at our tributary site, Cocalaco. While it is possible our results could be driven by non-adaptive variation in maternal investment, our results are a necessary initial step demonstrating size- and age-dependence in offspring size in some populations of *Xiphophorus birchmanni.* Future work is needed to rule out non-adaptive explanations, and to test whether adaptive mechanisms could explain this pattern.

The differences among our sites in the relationship between maternal size and offspring size suggest new insights into the potential constraints on offspring size. First, we note that where the female size ranges of San Pedro and Cocalaco overlap, the offspring produced at Cocalaco tended to be larger than the offspring produced at San Pedro (Figure 4). That is, the largest females at Cocalaco are producing larger embryos than are females of the same size at San Pedro (the smallest females in that population). This suggests the small females at San Pedro could produce larger offspring, as do their counterparts at Cocalaco. This supports the idea that the general pattern of size-dependence is not driven by a physical constraint (e.g. vent length) on offspring size.

One surprising outcome of our study was that our measure of female condition did not explain any additional variance in offspring size (beyond the variance explained by female size and age). We found that condition varied with female size, age, and site, and that there were significant interaction terms among these factors. Despite the fact that these factors are interrelated, our assay of female lipid stores was not related to offspring size in this species. It may be that differences in female condition affect other reproductive traits, such as the interbrood interval [42]. Further research is needed on the general role of resource availability in shaping offspring size and number in livebearers [4]–[6].

Although maternal size is the best predictor of offspring size, we found that female size and age are strongly related. Therefore, we are unable to distinguish whether the pattern of increased offspring size is due to a mechanism associated with maternal size or with age. It is possible that a size-dependent constraint on maternal physiology limits the offspring size that can be produced by smaller females. Although this mechanism was proposed by Sakai and Harada [30] to explain size-dependence in offspring investment, their model was inspired by observations of seed size in plants. There is little or no evidence suggesting that such a constraint exists for fish, although it is theoretically possible; further research on the physiology of egg provisioning could yield surprising insights.

Another possibility is that density-dependent sibling competition explains the correlation between maternal size and offspring size. As larger females are more fecund, density-dependent competition or survival is expected to be most important for offspring of larger females [29]. Although density-dependent egg mortality has been shown to occur in nests of sand gobies [43], little is known about possibility of density-dependent survival or competition for space among siblings in livebearers, as density-dependent processes are very difficult to study prior to birth. Again, more research is needed to determine the contribution of within-brood density-dependence to observed variation in swordtail offspring sizes.

Two recent theories provide alternative adaptive explanations for size- and age-dependent offspring investment [14]–[15]. One predicts that maternal size will be positively related to offspring size if larger offspring develop more slowly, and smaller females have a greater risk of mortality. These two factors interact to favor smaller, faster-developing embryos in smaller females [15]. If swordtail females experience size-dependent morality, and if development time is positively associated with egg size (as in other fish [26]), then this mechanism could explain the positive relationship between offspring size and maternal size. An alternative explanation is that older females are favored to increase both offspring size and number. Theory predicts age-dependence in offspring size is adaptive in species where total reproductive effort is costly to maternal survival [14], as long as resources are not limiting. Although our data cannot directly differentiate between these theories, and cannot exclude non-adaptive mechanisms, our results are consistent with both of these adaptive explanations. Our findings motivate future work testing the key features of these models to determine if they differ among our study populations.

While it is somewhat unsatisfying that our data do not allow us to test the size-dependent investment predictions in Kindsvater et al. [14] and Jørgensen et al. [15], these theories provide some general insights into the mechanisms that could explain our data and guide future research. Specifically, both theories invoke an indirect effect of female mortality on the optimal maternal investment strategy. The former theory also points out that both the degree to which reproduction is costly, and the shape of the offspring fitness function, will influence age-dependent variation in offspring size. This suggests that further investigation of the indirect effects of mortality risk on reproductive traits could be useful.

The importance of size- or age-dependent investment in offspring size depends on the contribution of offspring size to offspring fitness. Increased size at birth is generally thought to increase survival, competitive ability, or growth in the stages following independence from the female [5] [44]–[46]. However, much is still unknown about the rate at which fitness increases with size, or why it does so [21] [45]. Although our results suggest that maternal influences on offspring size can generate meaningful variation in this trait, it is only a first step towards the loftier goal of understanding the ecological and evolutionary consequences of this pattern. A fruitful next step would be to examine whether female size and age structure affect population growth rate. Studies of fish population dynamics have shown that, in some species, populations of older, larger females have a higher reproductive rate than populations of younger females [9]. While most research on this pattern focuses on the management implications for harvested fish stocks [9] [13], this phenomenon potentially has general consequences for our understanding of population processes. If we assume that larger swordtail offspring have increased survival, then it is possible that female age structure contributes through this pathway to population growth and evolution in our study populations. Our study shows that the relationship between maternal size and offspring size is also sensitive to the overall range of female sizes. This result motivates further investigation into the specific causes and consequences of this maternal effect on offspring size and fitness.

## Supporting Information

Figure S1 **Size, age, and condition of mature females are positively related.** Data are pooled across sites and years; to indicate depth, the point color shifts from red to black.
(TIF)
