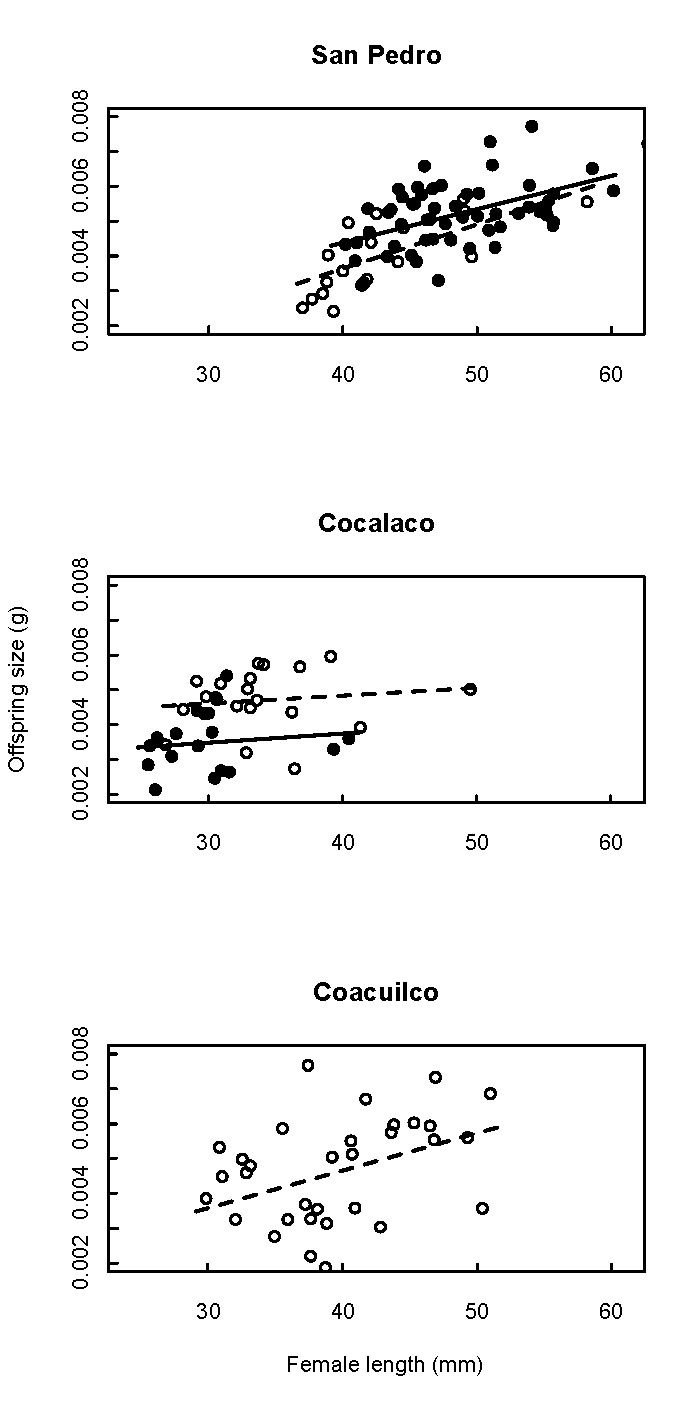# Correction: Maternal Size and Age Shape Offspring Size in a Live-Bearing Fish, *Xiphophorus birchmanni*

**DOI:** 10.1371/annotation/e8d4f506-c582-4413-9eb9-2b1b49ad1c81

**Published:** 2013-10-11

**Authors:** Holly K. Kindsvater, Gil G. Rosenthal, Suzanne H. Alonzo

In Figure 3, the numbers of the y-axis are in hundredths rather than in thousandths. Please see the corrected Figure 3 here: 

**Figure:**