# Supplementary figures and images for: Maternal Size and Age Shape Offspring Size in a Live-Bearing Fish, Xiphophorus birchmanni
Source: PLoS One. 2012 Nov 6;7(11):e48473. doi: 10.1371/journal.pone.0048473 (PMC3491049; doi:10.1371/journal.pone.0048473)

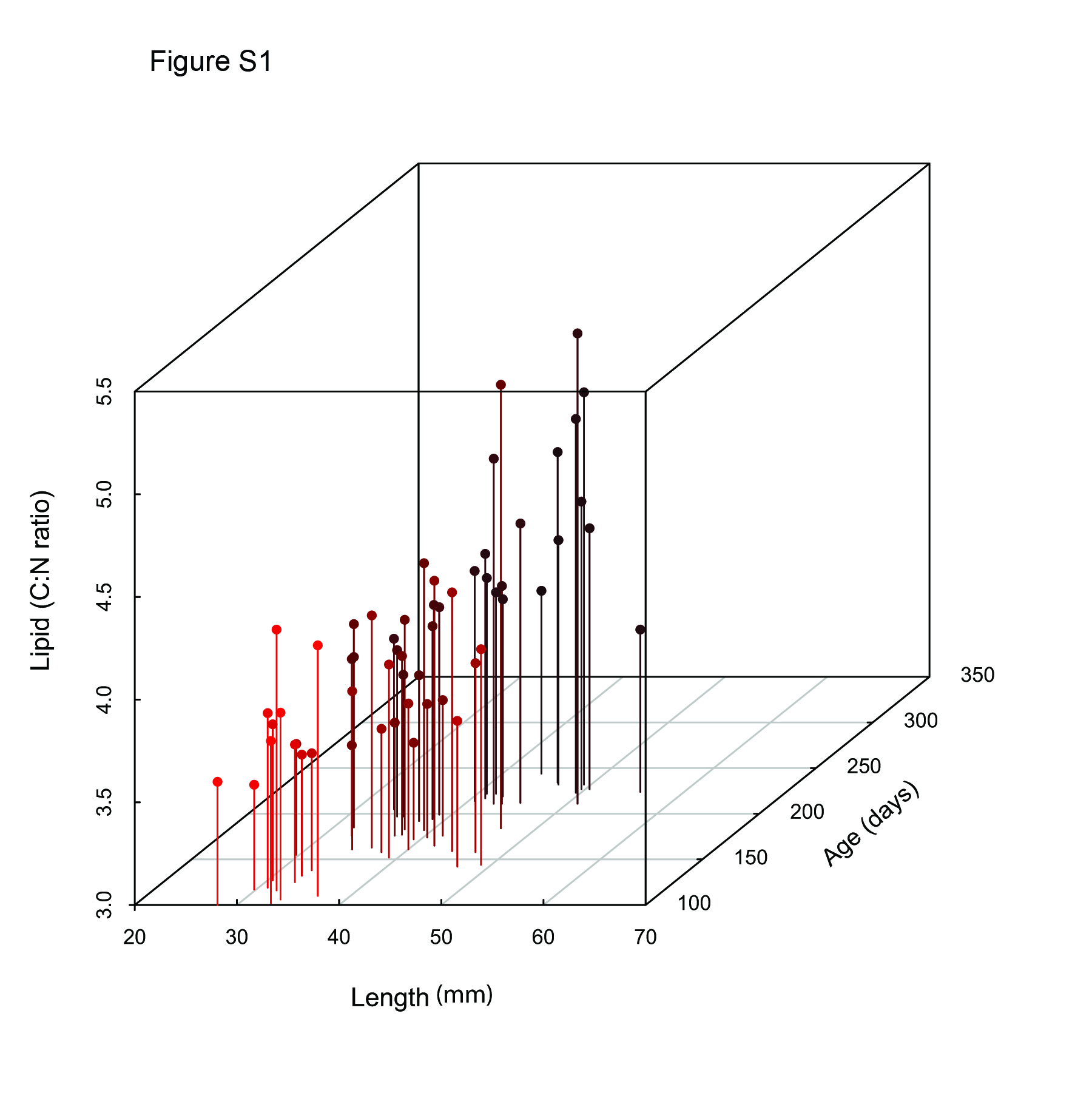

Supplement: Figure S1 — Size, age, and condition of mature females are positively related. Data are pooled across sites and years; to indicate depth, the point color shifts from red to black. (TIF) [file pone.0048473.s001.tif]
